# Association of Four Nutritional Scores With All-Cause and Cardiovascular Mortality in the General Population

**DOI:** 10.3389/fnut.2022.846659

**Published:** 2022-03-30

**Authors:** Heze Fan, Yuzhi Huang, Haoxuan Zhang, Xueying Feng, Zuyi Yuan, Juan Zhou

**Affiliations:** ^1^Cardiovascular Department, First Affiliated Hospital of Xi'an Jiaotong University, Xi'an, China; ^2^Key Laboratory of Environment and Genes Related to Diseases, Ministry of Education, Xi'an, China; ^3^Department of Bioengineering, Southwest Jiaotong University, Chengdu, China; ^4^Department of Biology, Georgia State University, Atlanta, GA, United States

**Keywords:** malnutrition, nutritional scores, all-cause death, cardiovascular death, general population

## Abstract

**Background and Aims:**

Malnutrition is a well known risk factor for adverse outcomes in patients with cancer, cardiovascular disease (CVD) and chronic kidney disease, but epidemiological evidence on its relationship with the long-term risk of all-cause mortality and cardiovascular death is limited.

**Methods:**

A total of 20,116 adults from the United States National Health and Nutrition Examination Survey 2007–2014 were enrolled. The Geriatric Nutritional Risk Index (GNRI), Prognostic Nutritional Index (PNI), Controlling Nutritional Status (CONUT) score, and Triglycerides (TG) × Total Cholesterol (TC) × Body Weight (BW) Index (TCBI) were calculated at baseline. Cox regression and the Kaplan–Meier analysis were conducted when participants were divided into three groups according to the tertiles of objective nutritional scores. Restricted cubic spline was performed to further explore the shape of the relationship between all-cause mortality, cardiovascular death, and nutritional scores. In addition, the area under the curve (AUC), continuous net reclassification improvement (NRI), and integrated discrimination improvement (IDI) were conducted to assess which nutritional scores have the greatest predictive value for all-cause death and cardiovascular death in the general population.

**Results:**

The cumulative incidence of all-cause death and cardiovascular death was significantly higher in participants with a higher CONUT score, lower GNRI, and lower PNI. TCBI showed the worst performance on grading and risk assessment. After adjusting confounding factors, the lowest PNI and GNRI tertile and highest COUNT score were independently and significantly associated with increased risk of all-cause death (all *P* < 0.01) and cardiovascular death (all *P* < 0.05) analyzed by a multivariate Cox regression model. An L-shaped association between the HR (hazard ratio) of all-cause mortality and nutritional scores (GNRI, PNI and TCBI) was observed in the overall populations. In addition, the PNI had the highest predictive value for all-cause mortality [AUC: 0.684, 95% confidence interval (CI): 0.667–0.701] and cardiovascular death (AUC: 0.710, 95% CI: 0.672–0.749) in the general population compared with other nutritional scores.

**Conclusion:**

The poorer the nutritional status of the general population, the higher the all-cause mortality and cardiovascular mortality. The PNI score may provide more useful predictive values than other nutritional scores.

## Introduction

Malnutrition is a prevalent problem in patients with chronic diseases, such as cancer, end-stage renal diseases (ESRD), coronary chronic total occlusion (CTO) (1–3). It is associated with higher complications, increased mortality, and length of hospitalization (4, 5). In addition, malnutrition can interfere with wound healing by delaying the healing response and was associated with poor cognitive development (6, 7). Therefore, nutrition management and assessment are essential for patients at risk of malnutrition.

However, it is difficult to comprehensively evaluate the nutritional status because malnutrition is affected by many factors (8). At present, four objective nutritional scores have been used in previous studies, including Geriatric Nutritional Risk Index (GNRI) (9), prognostic Nutritional Index (PNI) (10), Triglycerides (TG) × Total Cholesterol (TC) × Body Weight (BW) Index (TCBI) (11), controlling nutritional status (CONUT) score (12). These scores include two or three of the following elements: albumin, lymphocytes count, TC, TG, and body weight. Previous studies have demonstrated that the GNRI, PNI, COUNT, and TCBI have significant prognostic value for the mortality or adverse events of patients with a wide range of cardiovascular disease (CVD) (11, 13–15). The poor nutritional status assessed by these nutritional indexes is significantly associated with the poor clinical outcome of patients. According to a recent study, compared with PNI and TCBI, GNRI had the greatest incremental value in predicting mortality after acute myocardial infarction (16).

However, epidemiological evidence on which score is more effective in predicting all-cause mortality and cardiovascular death is limited. In addition, the relationship between these four nutritional scores and all-cause and cardiovascular mortality remains elusive in the general population. Therefore, we used the data from the National Health and Examination Survey (NHANES) to address the knowledge gap.

## Materials and Methods

### Study Population

**Figure 1:**
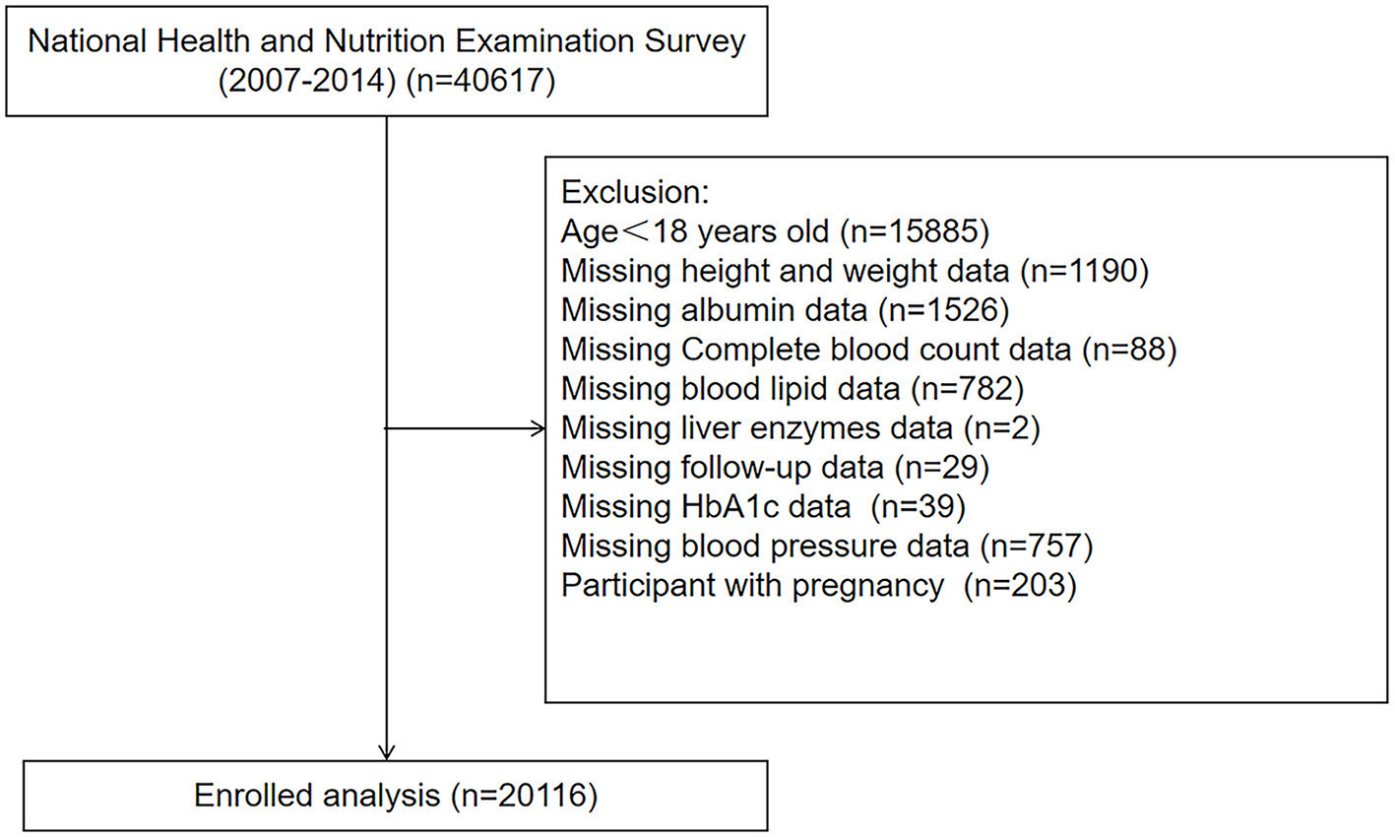
The flow chart of participant selection.

We analyzed data from the National Health and Nutrition Examination Survey (NHANES) between the period of 2007–2014, a nationwide cross-sectional survey conducted by the Center for Disease Control and Prevention (CDC) in the United States to assess the health status of US citizens (17). Participants with age < 18, pregnancy, and those without complete medical records were excluded. Finally, a total of 20,116 participants were enrolled in our study (Figure 1).

### Exposure

Serum albumin levels were measured using the bichromatic digital endpoint method on a DxC800. Lymphocyte counts were obtained from the whole blood using the Coulter method. The measurement of serum TG and TC was performed with enzymatic assays. Height and Weight were measured at the Mobile Examination Center (MEC) examination. If TG was ≤400 mg/dL, missing low-density lipoprotein cholesterol (LDL-C) data was computed by the Friedewald formula (18). The GNRI was calculated by using the following formula: GNRI = [1.489 × serum albumin (g/l)] +[41.7 × weight (kg)/ideal body weight (kg)]. The calculation of the ideal body was as follows: 22 × square of height because of its validity. The ratio of weight-to-ideal body weight was set to 1 if the actual body weight exceeded the ideal body weight (3). The PNI was defined by the following formula: PNI = serum albumin (g/L) + 5 × total lymphocyte count (10^9^/L). The TCBI was calculated using the formula: serum level of TG (mg/dL) × TC (mg/dL) × body weight (kg)/1000. The CONUT score was described in Supplementary Table 1. Nutritional scores (including GNRI, PNI, TCBI) were divided into three groups according to the tertiles: low (GNRI < 102.75), intermediate (GNRI: 102.75–107.22), and high (GNRI > 107.22) GNRI groups; low (PNI < 51), intermediate (PNI: 51–55), and high (PNI > 55) PNI groups; and low (TCBI < 1,211.19), intermediate (TCBI: 1,211.19–2,421.71), and high (TCBI > 2,421.71) TCBI groups. For the COUNT score, a score of 0 was considered normal nutritional status, scores of 1 to 2 were considered mild to moderate malnutrition, and scores of ≥3 were considered severe malnutrition.

### Covariates

With the use of standardized questionnaires, participants provided information on age, gender, race, smoking, drinking, medical history (hypertension, diabetes, and CVD), and medication use. Alanine aminotransferase (ALT), aspartate aminotransferase (AST), uric acid (UA), glycosylated hemoglobin (HbA1c), and creatinine (CRE) were measured by standard methods. The details of laboratory methodology are available at https://wwwn.cdc.gov/Nchs/Nhanes/2013-2014/BIOPRO_H.htm. Body mass index (BMI) was calculated using the following equation: body weight (kg)/ the square of height (m^2^). The estimated glomerular filtration rate (eGFR) was computed by Modification of Diet in Renal Disease formula (19). The race was classified as non-Hispanic white, non-Hispanic black, Mexican American, other Hispanic, or others. CVD history was defined as self-reported congestive heart failure, coronary heart disease, angina pectoris, heart attack, and stroke. Participants were considered as hypertensives if they were taking antihypertensive medications, had the average systolic blood pressure (SBP) exceeding 140 mmHg, or the average diastolic blood pressure (DBP) exceeding 90 mmHg. The mean systolic and diastolic blood pressures were calculated from up to four readings obtained in a seated position and using sphygmomanometers. Diabetes was defined as fasting glucose > 7 mmol/L or glycated hemoglobin A1c ≥6.5% or the usage of hypoglycemic drugs or history of diabetes.

### Outcomes

Outcomes of our study mainly were all-cause and cardiovascular mortality. Mortality status was obtained by linkage to the National Death Index by December 31, 2015. These mortality files are available for online access (https://www.cdc.gov/nchs/datalinkage/mortality-public.htm). Cardiovascular mortality in our study was defined according to the International Classification of Diseases, 10th Clinical Modification (ICD-10) System codes (I00–I09, I11, I13, I20–I51, I60–I69) (20).

### Statistical Analysis

Continuous variables are expressed as median with interquartile range. Categorical variables are expressed as frequencies and percentages. Depending on the nature of data, Chi-square, ANOVA, or Kruskal-Wallis H-test were performed to detect subgroup differences. The initial confounding factors were selected based on previous studies, data availability, and established associations (20–25). If these factors changed the estimates of PNI on all-cause mortality or cardiovascular death by more than 10% or were significantly associated with all-cause mortality or cardiovascular death after adjustment for sociodemographic factors (age, gender, and race), they were included as the covariates in the Cox regression analysis (26). Finally, we excluded platelet count and lipid-lowering drugs. Three sets of Cox regression models were constructed to evaluate the association of nutritional scores with all-cause and cardiovascular mortality. Model 1 only included nutritional scores. Model 2 was adjusted for age, sex, and race. Model 3 was adjusted for age, sex, race, BMI, Smoking, Drinking, hypertension, diabetes, HDL-C, LDL-C, SBP, DBP, eGFR, AST, ALT, UA, HbA1c, CVD, hypotensive drugs, and hypoglycemic drugs. Kaplan–Meier method was used to perform the analysis of the time-to-event data, and the log-rank test was used to compare the differences between each group. Restricted cubic splines (RCS) were applied using the R package “rms” based on the Cox proportional hazards models further to explore the relationship between the nutritional scores and endpoints. Receiver operating characteristic (ROC) curve analyses were performed using the R package “pROC” to compare these four nutritional indexes in predicting all-cause and cardiovascular mortality. The differences in the area under the curve (AUC) between two ROC curves were analyzed using DeLong test. Moreover, the continuous net reclassification improvement (NRI) and integrated discrimination improvement (IDI) were calculated using the R package “PredictABEL.” Subgroup analyses were conducted to evaluate whether the results between mortality and PNI were modified by age, gender, diabetes, hypertension, and cardiovascular diseases based on the fully adjusted multivariable regression model with interactions between PNI and stratified covariates. A *P* value < 0.05 was considered statistically significant. All statistical analyses were conducted using R software (version 4.1).

## Results

### Baseline Characteristics

**Figure 2:**
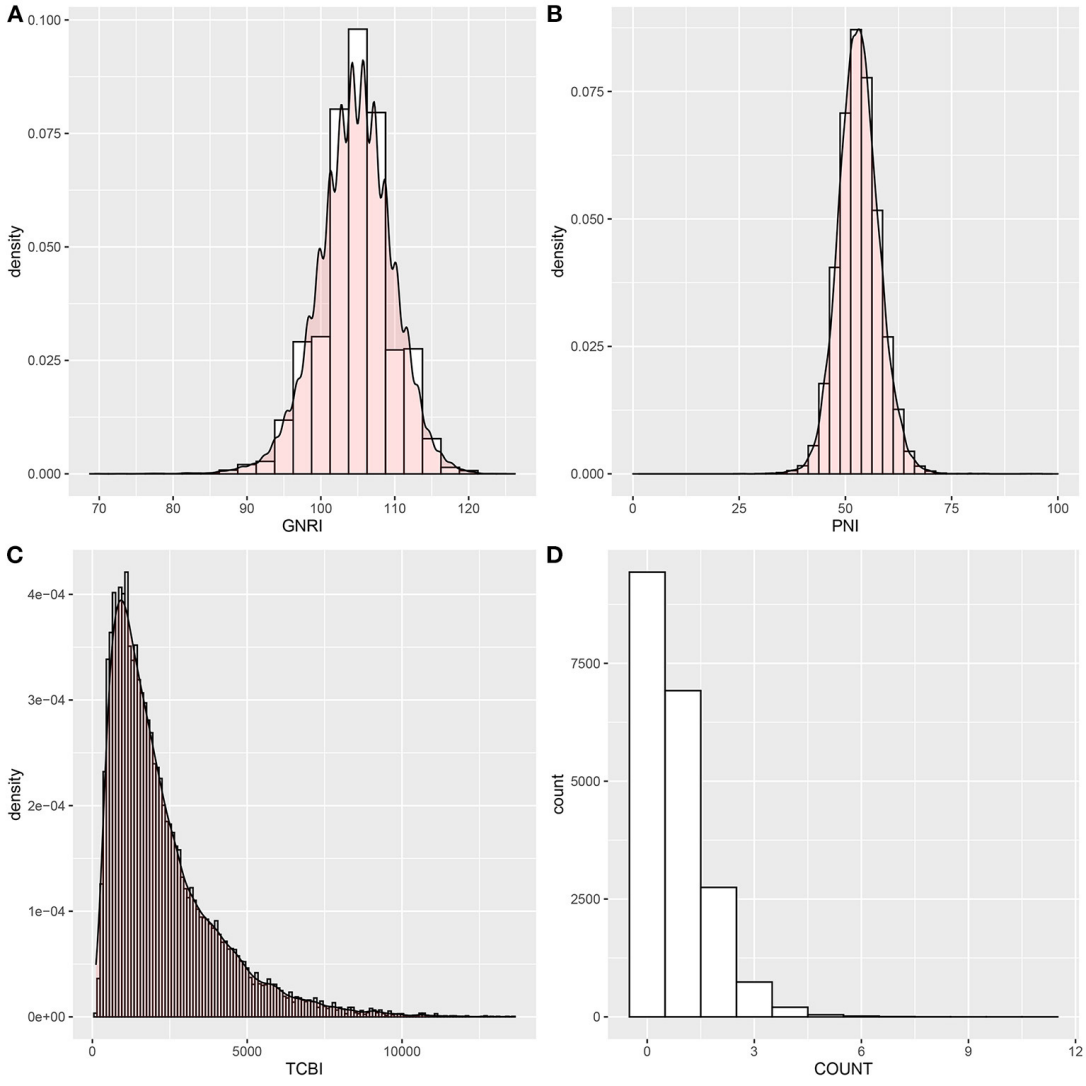
Histograms show the population distribution of nutritional scores. **(A)** GNRI; **(B)** PNI; **(C)** TCBI; **(D)** COUNT score. PNI, Prognostic Nutritional Index; GNRI, Geriatric Nutritional Risk Index; CONUT score, Controlling Nutritional Status score; TCBI, Triglycerides × Total Cholesterol × Body Weight Index.

The baseline characteristics of the study population according to PNI tertiles are shown in Table 1. A total of 20,116 participants were enrolled in this study, 1,188 patients died for various reasons, and 18,928 survived. The lowest tertile (PNI < 51) group had lower GNRI and TCBI and higher COUNT score (*p* < 0.001). In addition, hypertension, diabetes, and CVD were more common in the lowest tertile (PNI < 51) group (*p* < 0.001). All variables in the Table 1 were statistically different across participants in different PNI tertiles (all *p* < 0.001). The distributions of the GNRI, PNI, TCBI, and COUNT score are shown in Figure 2.

**Table 1:** Baseline characteristics.

	**PNI**			
**Variables**	** <51 (*n* = 6,074)**	**51–55 (*n* = 7,704)**	**>55 (*n* = 6,338)**	***P*-value**
Age (years)	57.0 (42.0–71.0)	47.0 (32.0–62.0)	39.0 (26.0–55.0)	<0.001
Gender, male, *n* (%)	2,585 (42.6%)	3,663 (47.5%)	3,598 (56.8%)	<0.001
Hypertension, *n* (%)	3,019 (49.7%)	2,966 (38.5%)	2,059 (32.5%)	<0.001
Diabetes, *n* (%)	1,260 (20.7%)	1,125 (14.6%)	766 (12.1%)	<0.001
Race, *n* (%)				<0.001
Mexican American	702 (11.6%)	1,248 (16.2%)	1,090 (17.2%)	
Non-Hispanic white	2,843 (46.8%)	3,332 (43.3%)	2,731 (43.1%)	
Non-Hispanic black	1,518 (25.0%)	1,540 (20.0%)	1,097 (17.3%)	
Other Hispanic	555 (9.1%)	831 (10.8%)	675 (10.7%)	
Other races	456 (7.5%)	753 (9.8%)	745 (11.8%)	
Hypotensive drugs, *n* (%)	2,129 (35.1%)	1,915 (24.9%)	1,161 (18.3%)	<0.001
Hypoglycemic drugs, *n* (%)	870 (14.3%)	773 (10.0%)	491 (7.7%)	<0.001
Cardiovascular disease, *n* (%)	954 (15.7%)	677 (8.8%)	383 (6.0%)	<0.001
Smoking, *n* (%)	2,665 (43.8%)	3,342 (43.4%)	2,965 (46.8%)	<0.001
Drinking, *n* (%)	3,816 (62.8%)	5,090 (66.1%)	4,223 (66.6%)	<0.001
BMI, kg/m^2^	28.6 (24.7–33.5)	27.5 (23.9–31.9)	26.9 (23.4–31.1)	<0.001
SBP, mmHg	123.0 (111.0–136.0)	119.0 (110.0–131.0)	119.0 (110.0–129.0)	<0.001
DBP, mmHg	69.0 (61.0–77.0)	70.0 (63.0–77.0)	71.0 (63.0–77.0)	<0.001
Lymphocyte count (10^∧^9/L)	1.6 (1.3–1.9)	2.3 (2.0–2.7)	3.1 (2.6–3.6)	<0.001
Total cholesterol, mg/dL	184.0 (159.0–211.0)	188.0 (163.0–216.0)	191.0 (164.0–220.0)	<0.001
Triglyceride, mg/dL	108.0 (74.0–159.0)	113.0 (76.0–173.0)	127.0 (83.0–195.0)	<0.001
HDL, mg/dL	52.0 (43.0–63.0)	51.0 (42.0–62.0)	49.0 (41.0–59.0)	<0.001
LDL, mg/dL	105.1 (83.0–128.0)	108.0 (86.0–132.4)	109.6 (87.0–134.9)	<0.001
AST, U/L	22.0 (19.0–27.0)	23.0 (20.0–27.0)	24.0 (20.0–28.0)	<0.001
ALT, U/L	19.0 (15.0–25.0)	21.0 (16.0–28.0)	22.0 (17.0–30.0)	<0.001
Albumin, g/L	40.0 (38.0–42.0)	43.0 (41.0–44.0)	45.0 (43.0–47.0)	<0.001
UA, umol/L	315.2 (261.7–374.7)	315.2 (261.7–368.8)	327.1 (273.6–386.6)	<0.001
eGFR, mg/min/1.73 m^2^	81.1 (65.0–97.9)	86.9 (71.9–104.3)	89.4 (75.4–104.9)	<0.001
HbA1c, %	5.6 (5.3–6.0)	5.5 (5.2–5.8)	5.4 (5.2–5.8)	<0.001
GNRI	101.3 (98.3–102.7)	105.7 (102.7–107.2)	108.7 (105.7–111.7)	<0.001
TCBI	1,604.8 (964.7–2,638.6)	1,698.9 (987.6–2,889.5)	1,916.0 (1,060.3–3,314.9)	<0.001
COUNT score, ≥3, *n* (%)	859 (14.1%)	137 (1.8%)	20 (0.3%)	<0.001

*Values are given as median and interquartile range or numbers and percentages*.

*PNI, Prognostic Nutritional Index; BMI, body mass index; SBP, systolic blood pressure; DBP, diastolic blood pressure; HDL, High-density lipoprotein; LDL, Low-density lipoprotein; AST, aminotransferase; ALT, alanine aminotransferase; UA, uric acid; HbA1c, glycosylated hemoglobin; eGFR, estimated glomerular filtration rate; GNRI, Geriatric Nutritional Risk Index; CONUT score, Controlling Nutritional Status score; TCBI, Triglycerides × Total Cholesterol × Body Weight Index*.

### The Relationship of Four Nutritional Scores With All-Cause and Cardiovascular Mortality

**Figure 3:**
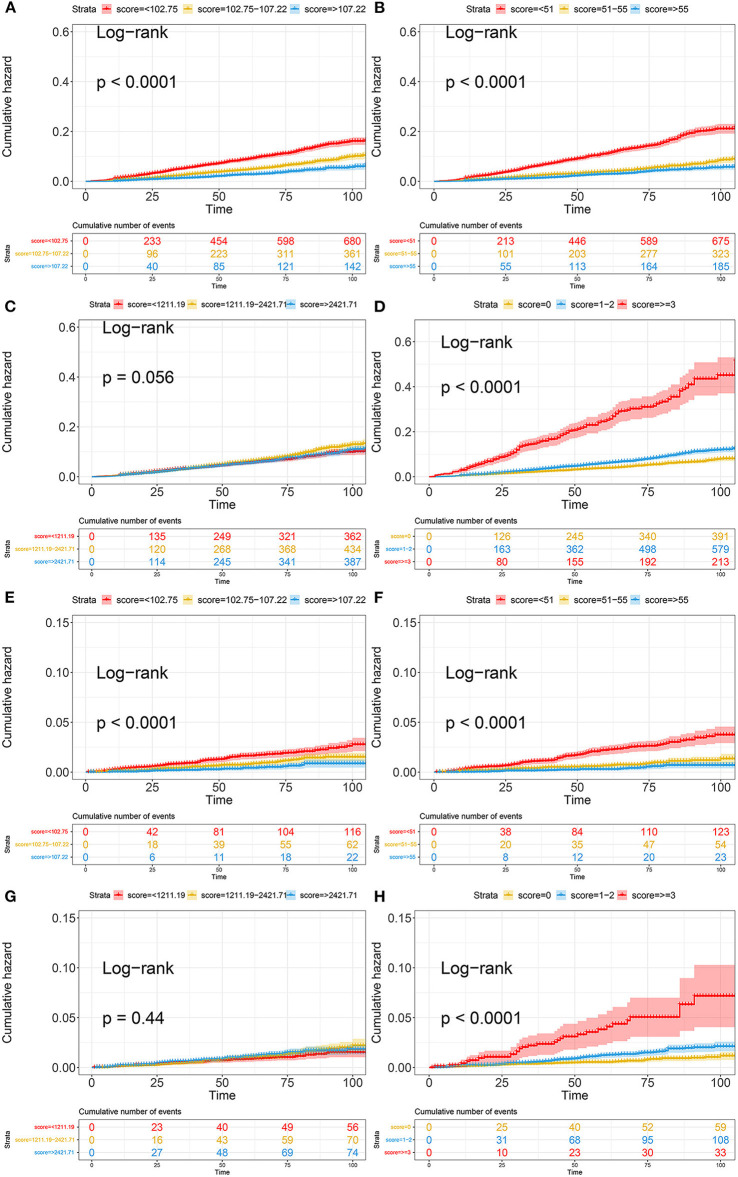
Kaplan-Meier curves of all-cause death and cardiovascular death based on four nutritional scores. **(A)** Kaplan-Meier curves of all-cause death categorized by GNRI; **(B)** Kaplan-Meier curves of all-cause death categorized by PNI; **(C)** Kaplan-Meier curves of all-cause death categorized by TCBI; **(D)** Kaplan-Meier curves of all-cause death categorized by COUNT score; **(E)** Kaplan-Meier curves of cardiovascular death categorized by GNRI; **(F)** Kaplan-Meier curves of cardiovascular death categorized by PNI; **(G)** Kaplan-Meier curves of cardiovascular death categorized by TCBI; **(H)** Kaplan-Meier curves of cardiovascular death categorized by COUNT score. PNI, Prognostic Nutritional Index; GNRI, Geriatric Nutritional Risk Index; CONUT score, Controlling Nutritional Status score; TCBI, Triglycerides × Total Cholesterol × Body Weight Index.

Kaplan–Meier curves illustrated the incidence of all-cause death and cardiovascular death in the general population (Figure 3). Overall, the cumulative incidence of all-cause mortality and cardiovascular death was significantly higher in patients with a lower PNI and GNRI or higher COUNT score. TCBI showed the worst performance on grading and risk assessment for all-cause death (log-rank test, *p* = 0.056) and cardiovascular death (log-rank test, *p* = 0.44) in the general population (Figures 3C,G). In addition, PNI showed superior performance on grading and risk assessment to other nutritional scores.

According to the nutritional scores calculated by each equation as the categorical variable grouped previously mentioned, we performed the Cox regression hazard models. Cox regression analysis of nutritional scores with all-cause mortality and cardiovascular death in the overall population is shown in Tables 2, 3. In the univariate and multivariate Cox proportional hazards analysis, participants with the lowest GNRI (< 102.75) and PNI (< 51) had increased risks of all-cause death and cardiovascular death. In addition, the intermediate GNRI (102.75–107.22) and PNI (51–55) were also associated with the incidence of adverse events for all-cause death and cardiovascular death in univariate analysis. For COUNT score, the highest and intermediate groups were associated with increased risk for all-cause mortality and cardiovascular death in Model 1, 2, and 3. For TCBI, although the lowest score was associated with increased all-cause mortality in Model 2 and 3, the positive effect size was non-significant in Model 1. Conversely, the intermediate TCBI score was associated with increased all-cause mortality in Model 1. Furthermore, we also found that TCBI was non-significantly associated with cardiovascular death.

**Table 2:** Cox regression analysis of nutritional scores with all-cause mortality.

**Nutritional score**		**Model 1**		**Model 2**		**Model 3**	
		**HR (95% CI)**	***p***	**HR (95% CI)**	***p***	**HR (95% CI)**	***p***
GNRI	< 102.75	3.09 (2.58–3.70)	<0.01	1.81 (1.51–2.17)	<0.01	1.81 (1.50–2.17)	<0.01
	102.75–107.22	1.72 (1.42–2.09)	<0.01	1.15 (0.95–1.39)	0.16	1.18 (0.97–1.44)	0.09
	>107.22	Reference		Reference		Reference	
PNI	< 51	3.86 (3.28–4.54)	<0.01	1.69 (1.43–2.00)	<0.01	1.79 (1.52–2.12)	<0.01
	51–55	1.42 (1.20–1.70)	<0.01	1.00 (0.84–1.20)	0.96	1.06 (0.89–1.27)	0.52
	>55	Reference		Reference		Reference	
TCBI	<1,211.19	1.01 (0.87–1.16)	0.95	1.29 (1.12–1.49)	<0.01	1.42 (1.19–1.69)	<0.01
	1,211.19–2,421.71	1.16 (1.01–1.33)	0.04	1.04 (0.90–1.19)	0.60	1.09 (0.94–1.26)	0.24
	>2,421.71	Reference		Reference		Reference	
COUNT	0	Reference		Reference		Reference	
	1–2	1.54 (1.36–1.75)	<0.01	1.35 (1.19–1.54)	<0.01	1.32 (1.14–1.53)	<0.01
	≥3	6.33 (5.36–7.48)	<0.01	2.92 (2.45–3.48)	<0.01	2.71 (2.20–3.33)	<0.01

*Data are presented as hazard ratios, 95% CI (confidence intervals), and P-value*.

*Model 1 adjusted for none*.

*Model 2 adjusted for age, sex, and race*.

*Model 3 adjusted for age, sex, race, BMI, Smoking, Drinking, hypertension, diabetes, HDL, LDL, SBP, DBP, eGFR, AST, ALT, UA, HbA1c, cardiovascular disease, hypotensive drugs, hypoglycemic drugs*.

**Table 3:** Cox regression analysis of nutritional scores with cardiovascular death.

**Nutritional score**		**Model 1**		**Model 2**		**Model 3**	
		**HR (95% CI)**	***p***	**HR (95% CI)**	***p***	**HR (95% CI)**	***p***
GNRI	< 102.75	3.45 (2.19–5.44)	<0.01	1.83 (1.16–2.91)	<0.01	1.77 (1.11–2.83)	0.02
	102.75–107.22	1.93 (1.19–3.13)	<0.01	1.20 (0.74–1.96)	0.46	1.24 (0.76–2.02)	0.39
	>107.22	Reference		Reference		Reference	
PNI	< 51	5.70 (3.65–8.89)	<0.01	2.11 (1.34–3.32)	<0.01	2.23 (1.41–3.52)	<0.01
	51–55	1.92 (1.18–3.13)	<0.01	1.27 (0.78–2.08)	0.33	1.36 (0.83–2.22)	0.22
	>55	Reference		Reference		Reference	
TCBI	<1,211.19	0.81 (0.57–1.15)	0.23	0.96 (0.67–1.36)	0.81	1.26 (0.82–1.95)	0.29
	1,211.19–2,421.71	0.98 (0.71–1.36)	0.89	0.82 (0.59–1.13)	0.23	0.94 (0.66–1.33)	0.72
	>2,421.71	Reference		Reference		Reference	
COUNT	0	Reference		Reference		Reference	
	1–2	1.89 (1.38–2.60)	<0.01	1.49 (1.08–2.05)	0.02	1.50 (1.04–2.15)	0.03
	≥3	6.41 (4.19–9.82)	<0.01	2.29 (1.46–3.58)	<0.01	2.31 (1.38–3.88)	<0.01

*Data are presented as hazard ratios, 95% CI (confidence intervals), and P-value*.

*Model 1 adjusted for none*.

*Model 2 adjusted for age, sex, and race*.

*Model 3 adjusted for age, sex, race, BMI, Smoking, Drinking, hypertension, diabetes, HDL, LDL, SBP, DBP, eGFR, AST, ALT, UA, HbA1c, cardiovascular disease, antihypertensive drugs, hypoglycemic drugs*.

**Figure 4:**
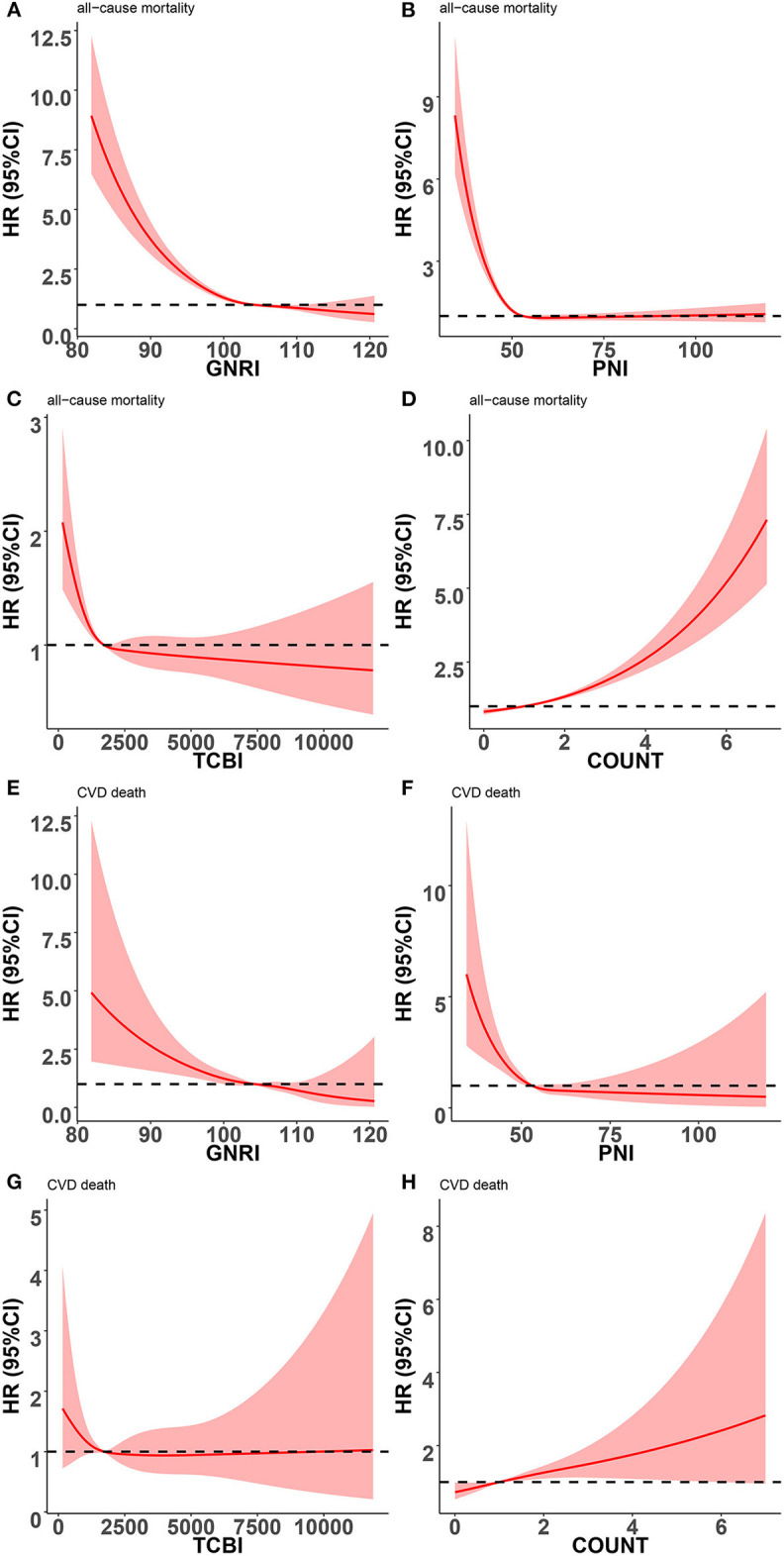
Restricted spline curves for the associations between four nutritional scores and adverse events in general population. Red lines represent the HR (hazard ratio), and red transparent areas represent the 95% confidence intervals. HR (95% CI) were all adjusted according to Model 3 in Cox analysis. **(A)** Association between GNRI and all-cause mortality; **(B)** Association between PNI and all-cause mortality; **(C)** Association between TCBI and all-cause mortality; **(D)** Association between COUNT score and all-cause mortality; **(E)** Association between GNRI and cardiovascular death; **(F)** Association between PNI and cardiovascular death; **(G)** Association between TCBI and cardiovascular death; **(H)** Association between COUNT score and cardiovascular death; PNI, Prognostic Nutritional Index; GNRI, Geriatric Nutritional Risk Index; CONUT score, Controlling Nutritional Status score; TCBI, Triglycerides × Total Cholesterol × Body Weight Index.

Restricted cubic splines were performed to further explore the associations of nutritional scores, which were treated as a continuous variable, with the HR (hazard ratio) of all-cause mortality and cardiovascular death after adjusting as Model 3 used in Cox analysis. An L-shaped relationship between the HR of all-cause mortality and nutritional scores (GNRI, PNI, and TCBI) was indicated in the overall populations (Figure 4). For GNRI, the HR sharply decreased until it reached ~104; thereafter, the HR tended to decrease slowly (Figure 4A). For PNI, the HR sharply decreased until it reached ~52–53; thereafter, the HR tended to the horizontal line with HR = 1 (Figure 4B). For TCBI, the HR sharply decreased until it reached ~1,689; thereafter, the HR tended to decrease slowly and the TCBI gradually showed a protective role but no statistical significance (Figure 4C). The higher the COUNT score, the worse the nutritional status of participants. For the COUNT score, the HR slowly increased until it reached ~2; thereafter, the HR tended to increase sharply (Figure 4D). We also found similar relationships between nutritional scores and cardiovascular death in the general population (Figure 4). However, the non-linear relationship between nutrition scores and cardiovascular death was weakened. In addition, no apparent correlation was found between the TCBI and cardiovascular death (Figure 4G).

### Comparative Analysis of Four Nutritional Scores in Predicting All-Cause and Cardiovascular Mortality

**Figure 5:**
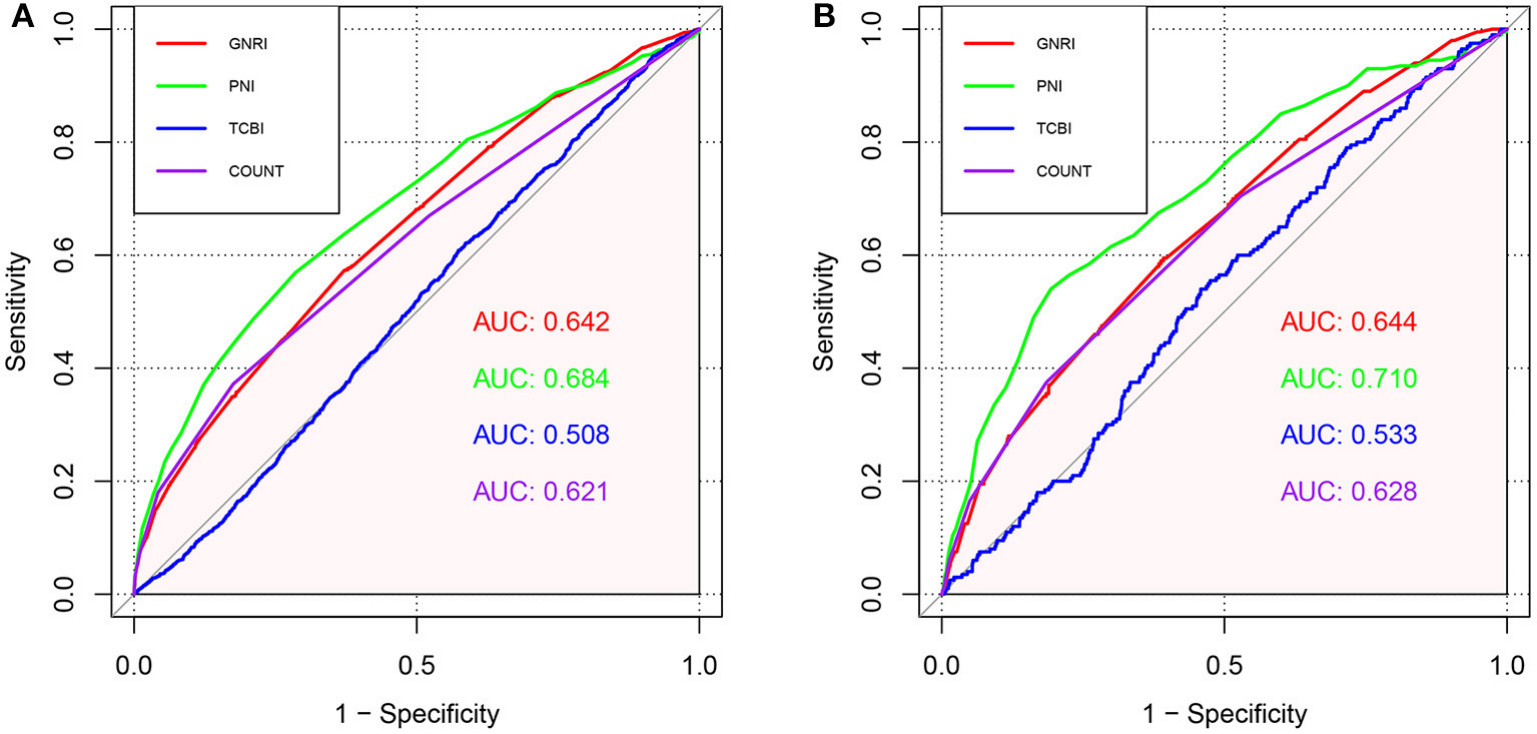
ROC curves for predicting adverse events in the general population. **(A)** ROC curves for predicting all-cause mortality plotted by the nutritional scores in general population. **(B)** ROC curves for predicting cardiovascular mortality plotted by the nutritional scores in general population. ROC, Receiver operating characteristic; AUC, area under the curve.

We conducted ROC curve analysis of the predictive models for all-cause death and cardiovascular death with the GNRI, PNI, TCBI, and COUNT score (Figure 5; Table 4). In terms of the AUC for all-cause mortality, the PNI score had significantly higher AUC [0.684, 95% confidence intervals (CI): 0.667–0.701), reference] than the other nutritional scores, whereas the GNRI (AUC 0.642, 95% CI: 0.626–0.659, *p* < 0.001) and the COUNT score (AUC 0.621, 95% CI: 0.604–0.638, *p* < 0.001) had similar AUC. The TCBI had the lowest AUC (0.508, 95% CI: 0.492–0.524, *p* < 0.001). We also obtained similar results in the AUC for cardiovascular death (Table 4).

**Table 4:** Comparisons of AUC, IDI, and NRI for GNRI, PNI, TCBI and COUNT.

**Models**	**NRI (95% CI)**	***P*-Value**	**IDI (95% CI)**	***P*-Value**	**AUC (95% CI)**	***P*-value**
**All-cause mortality**						
GNRI	−0.210 (−0.267, −0.154)	<0.001	−0.007 (−0.010, −0.004)	<0.001	0.642(0.626–0.659)	<0.001
PNI	Reference		Reference		0.684 (0.667–0.701)	Reference
TCBI	−0.532 (−0.588, −0.476)	<0.001	−0.031 (−0.035, −0.028)	<0.001	0.508 (0.492–0.524)	<0.001
COUNT	−0.176 (−0.234, −0.118)	<0.001	−0.003 (−0.006, −2e-04)	0.036	0.621 (0.604–0.638)	<0.001
**Cardiovascular death**						
GNRI	−0.485 (−0.615, −0.356)	<0.001	−0.004 (−0.005, −0.003)	<0.001	0.644 (0.606–0.682)	0.016
PNI	Reference		Reference		0.710 (0.672–0.749)	Reference
TCBI	−0.601 (−0.733, −0.470)	<0.001	−0.007 (−0.009, −0.005)	<0.001	0.533 (0.495–0.570)	<0.001
CPUNT	−0.490 (−0.621, −0.360)	<0.001	−0.004 (−0.005, −0.003)	<0.001	0.628 (0.589–0.668)	<0.001

*IDI, integrated discrimination improvement; NRI, net reclassification improvement*.

Additionally, NRI and IDI in all-cause mortality and cardiovascular death were assessed by comparing GNRI, TCBI, and COUNT to PNI (reference). Overall, compared with PNI, the reclassification of other nutritional scores performed worse both on all-cause mortality and cardiovascular death (Table 4). The NRI and IDI of GNRI (−0.210, *p* < 0.001 and −0.007, *p* < 0.001, NRI and IDI respectively), TCBI (−0.532, *p* < 0.001 and −0.031, *p* < 0.001, NRI and IDI respectively), and COUNT score (−0.176, *p* < 0.001 and −0.003, *p* = 0.036, NRI and IDI respectively) for the all-cause death were significantly inferior to PNI. In addition, the PNI remained incremental values for predicting the incidence of cardiovascular death.

### Stratification Analysis

Since PNI had the highest predictive value for all-cause death and cardiovascular death in the general population, we stratified the individuals by age, gender, hypertension, diabetes, and cardiovascular diseases to further observe the association between PNI and all-cause mortality, cardiovascular death. The PNI (divided by the median of 53) was further treated as a dichotomous variable in subgroup analysis.

**Figure 6:**
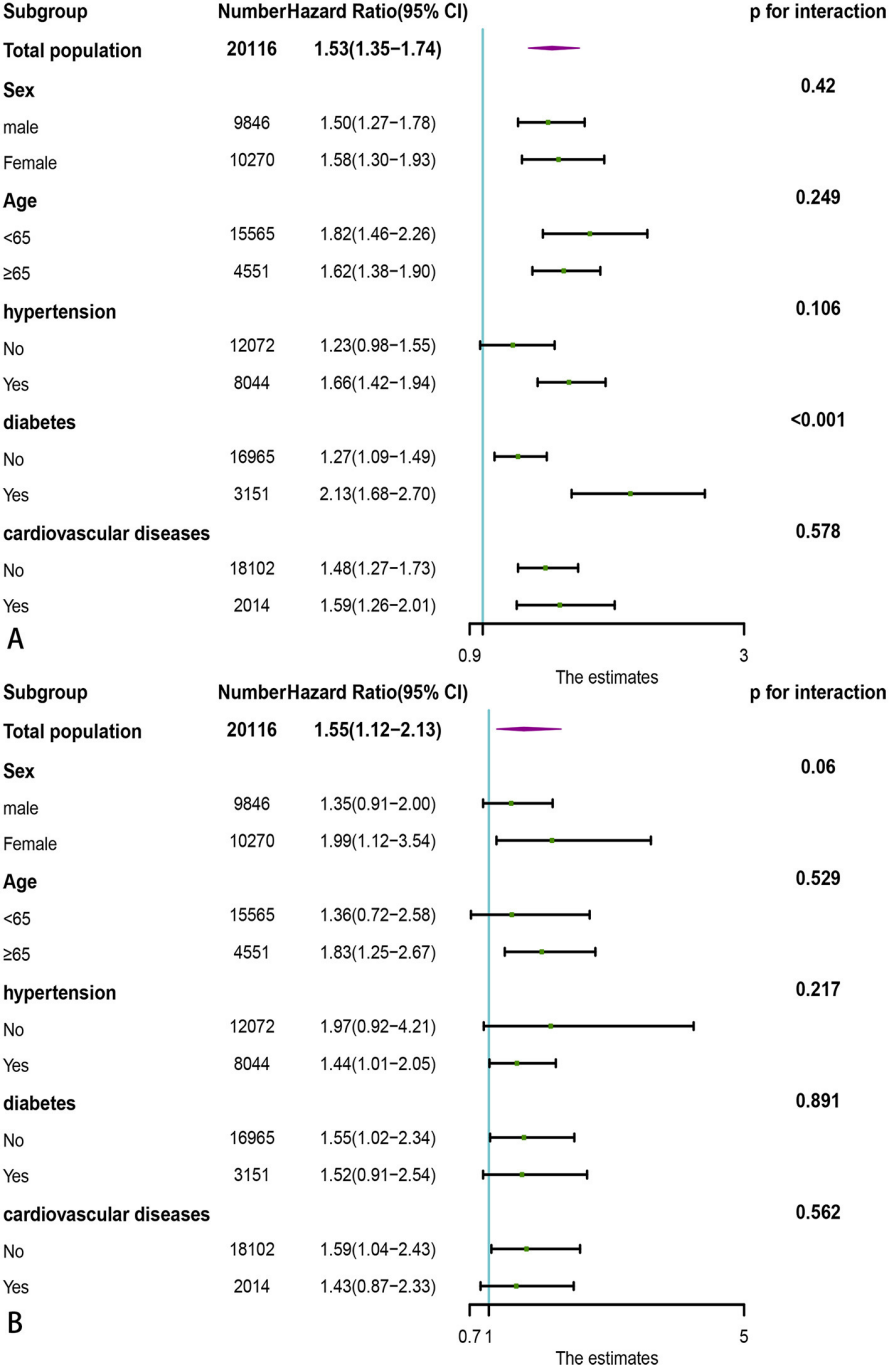
Subgroup analysis of association of PNI with adverse outcomes in the general population. **(A)** All-cause death; **(B)** Cardiovascular death. PNI, Prognostic Nutritional Index.

As shown in Figure 6A, lower PNI was found to be associated with increased risks of all-cause death in almost all the subgroups except for participants without hypertension (HR = 1.23, 95% CI: 0.98–1.55). In addition, lower PNI was also associated significantly with increased risks of cardiovascular death in total population (HR = 1.55, 95% CI: 1.12–2.13) and half of subgroups (Figure 6B), except for male (HR = 1.35, 95% CI: 0.91–2.00), age < 65 (HR = 1.36, 95% CI: 0.72–2.58), participants without hypertension (HR = 1.97, 95% CI: 0.92–4.21), participants with diabetes (HR = 1.52, 95% CI: 0.91–2.54) and participants with CVD (HR = 1.43, 95% CI: 0.87–2.33). We also found that the association between lower PNI and all-cause mortality was more pronounced in the diabetic population [HR = 2.13, 95% CI: 1.68–2.70, *p* for interaction < 0.001].

## Discussion

In the present study, the nutritional status was evaluated by the GNRI, TCBI, PNI, and COUNT score. We explored their association with all-cause mortality and cardiovascular death in the general population recruited from a nationally representative sample of the United States. The major conclusions are as follows: (1) Malnourished participants were at a higher risk of all-cause death and cardiovascular death. (2) We found an L-shaped relationship between nutritional scores (GNRI, PNI, and TCBI) and adverse outcomes in the general population. However, for COUNT score, the L-shaped association was the opposite. (3) Compared with other nutritional scores, the PNI had the highest predictive value in the general population. However, TCBI showed the worst performance on risk assessment and prediction. (4) The association between lower PNI and all-cause mortality was more pronounced in the diabetic population.

The PNI, which consists of serum albumin level and the total lymphocyte count, has been widely applied to predict adverse outcomes in various patients (14, 27–29). The main advantages of PNI compared to other nutrition scores are as follows. First, besides being a nutritional indicator, there is evidence that serum albumin regulates the body's inflammatory response and negatively correlates with C-reactive protein (CRP) levels (30, 31). The stabilization of inflammatory cytokines and oxidative stress markers is also an important role of albumin (32). Meanwhile, it is well known that low lymphocyte count (LLC) can reflect a poorly regulated immune response. In addition, LLC is a common phenomenon during the inflammatory reaction (33). The above evidence suggested that PNI could not only assess the nutritional status but also effectively reflect the inflammation and immune status of the body. Second, the predictive value of low albumin for adverse events has been reported in many studies (34, 35), and LLC is also associated with poor prognosis in patients with a wide variety of diseases (33, 36). Therefore, participants with low PNI were at extremely high risk of mortality. Third, compared with COUNT score using categorical variables, albumin and lymphocyte count are used as continuous variables to calculate PNI, which minimizes the loss of information and better reflects the nutritional status of the general population. Fourth, lymphocyte count is a more stable indicator of body composition during long-term follow-up. In contrast, the indicators (body weight, TC, and TG) used to calculate GNRI and TCBI are more susceptible to some factors such as age, diet, drugs, smoking, drinking, and lifestyle habits. Therefore, our study suggested that PNI might be the most effective indicator for predicting the adverse events of the general population among the four nutritional scores. In the subgroup analysis, the risk of all-cause death in diabetic patients with lower PNI was significantly higher than that in non-diabetic patients, which showed that malnutrition might play a more critical role in the mortality of diabetic patients. One possible explanation is that malnutrition may aggravate systemic inflammation in diabetic patients, leading to increased all-cause mortality (37, 38).

The GNRI, which considers both serum albumin levels and body weight, is also used to assess the nutritional status of patients and shows the predictive value for adverse outcomes (2, 3). The unique advantage of the GNRI is that the ratio of body weight to ideal body weight allows for a better reflection of the extent to which malnourished participants deviate from normal BMI, which can help assess short-term nutritional status. However, the weight may change substantially during long-term follow-up, especially in young adults, which limits its predictive value in the general population. Furthermore, it is essential to emphasize that body weight is influenced by fluid distribution in the body, which may make the measured weight of participants with edema higher than their actual weight (39). Therefore, GNRI may overestimate the nutritional status of this population.

The TCBI index, a novel nutritional metabolism index, is calculated from variables reflecting lipid metabolism measured from blood tests. Some studies showed that TCBI was a useful prognostic indicator in patients with a wide range of CVD (11, 40, 41). The most significant advantage of the TCBI is the simplicity of the calculation, which saves time and effort in caring for and treating ICU (intensive care unit) patients (40, 41). The calculation of TCBI simply requires multiplying three variables, while anyone who calculates GNRI or PNI needs to know the constant values and how to calculate ideal body weight. For CONUT, they need to know thresholds and scores for each indicator. In our study, TCBI was perhaps not an ideal tool in predicting adverse events in the general population. We think this may be caused by the reason that TC and TG cannot accurately reflect the nutritional status, inflammation level, and immune response of the body. On the one hand, the relationship between reduced TG and TC and poor nutritional status is currently not fully elucidated. On the other hand, as previously described, TC and TG are more prone to change due to various factors.

The COUNT score is also reported as an independent prognostic marker in patients with various malignancies (42, 43), acute heart failure (44), and coronary artery disease (45). The advantage of the COUNT score is that it incorporates the largest number of serum nutritional indicators. Compared with PNI, the COUNT score considers the influence of TC on nutritional status. Compared with GNRI and TCBI, lymphocyte count is included as a more stable indicator. However, our study demonstrated the COUNT score was lower than PNI in predicting all-cause mortality and cardiovascular death. We think this may be because the COUNT score treats serum albumin levels, total lymphocyte count, and TC as categorical variables, which is its greatest deficiency.

Malnutrition may decrease immunity and antioxidant capacity and increase inflammation and blood viscosity, which may lead to the occurrence of adverse outcomes. In our study, lower PNI (<51) and GNRI (<102.75) and higher COUNT score (>3) were significant independent predictors of all-cause mortality and cardiovascular death in the general population. The restricted cubic spline showed an L-shaped relationship between nutritional scores and adverse events, which also indicated that malnutrition significantly increased the risk of death. In addition, since PNI had the highest predictive value, we needed to focus more on the nutritional status assessed by PNI to reduce all-cause and cardiovascular mortality in the general population.

Despite the crucial findings being mentioned, our study has some limitations. First, the results were mainly applicable in the United States. Second, some covariates were self-reported using validated questionnaires, which might be affected by memory bias. Third, it is unclear whether nutritional status changes over time could influence the risk of all-cause mortality and cardiovascular death. Fourth, we only explored the association of nutritional scores with all-cause mortality and cardiovascular death but not with mortality related to other causes.

## Conclusion

In summary, we reported an association between all-cause mortality, cardiovascular death and four objective nutritional scores (GNRI, PNI, TCBI, and COUNT). Meanwhile, our study demonstrated that the PNI had the greatest predictive value for all-cause mortality and cardiovascular death in the general population.

## Data Availability Statement

The original contributions presented in the study are included in the article/Supplementary Material, further inquiries can be directed to the corresponding author/s.

## Ethics Statement

The studies involving human participants were reviewed and approved by National Center for Health Statistics. The patients/participants provided their written informed consent to participate in this study.

## Author Contributions

HF: conceptualization, project administration, writing—original draft, methodology, data analysis, and data collection. YH and HZ: methodology, data analysis, and data collection. XF: data curation and data collection. ZY: resources, funding acquisition, and writing—review and editing. JZ: supervision, project administration, data curation, writing—review and editing, and methodology. All authors contributed to the article and approved the submitted version.

## Funding

This study was supported by the National Key R&D Program of China (2021YFA1301200 and 2019YFA0802300), the Key Project of Research and Development Plan (2017ZDCXL-SF-02-04-01), and the Clinical Research Award of the First Affiliated Hospital of Xi'an Jiaotong University, China (No. XJTU1AF-CRF- 2016-004).

## Conflict of Interest

The authors declare that the research was conducted in the absence of any commercial or financial relationships that could be construed as a potential conflict of interest.

## Publisher's Note

All claims expressed in this article are solely those of the authors and do not necessarily represent those of their affiliated organizations, or those of the publisher, the editors and the reviewers. Any product that may be evaluated in this article, or claim that may be made by its manufacturer, is not guaranteed or endorsed by the publisher.
