# Supplementary material for: Association of Four Nutritional Scores With All-Cause and Cardiovascular Mortality in the General Population
Source: Front Nutr. 2022 Mar 30;9:846659. doi: 10.3389/fnut.2022.846659 (PMC9006821; doi:10.3389/fnut.2022.846659)
Supplement: Supplementary file 1 [file Table_1.DOCX]

Supplementary Material

# Supplementary Tables

| Table S1. Evaluation of COUNT score | | | | |
| --- | --- | --- | --- | --- |
| Parameter | Score |  |  |  |
| Serum albumin, g/L | ≥35 | 30-34 | 25-29 | <25 |
| Albumin score | 0 | 2 | 4 | 6 |
| Total cholesterol, mg/dl | ≥180 | 140-179 | 100-139 | <100 |
| Cholesterol score | 0 | 1 | 2 | 3 |
| Total lymphocytes, 10^9/L | ≥1.600 | 1.200-1.599 | 0.800-1.199 | <0.800 |
| Lymphocyte score | 0 | 1 | 2 | 3 |
